# Expression and prognostic analyses of the significance of STEAP1 and STEAP2 in lung cancer

**DOI:** 10.1186/s12957-022-02566-6

**Published:** 2022-03-28

**Authors:** Tianshu Liu, Xiaoxin Niu, Yanqing Li, Zekun Xu, Jie Chen, Geng Xu

**Affiliations:** 1 Department of Maternal and Child Health, School of Public Health, Cheeloo College of Medicine, Shandong University, Jinan, 250012 Shandong China; 2 Department of Thoracic Surgery, Heze Municipal Hospital, Heze, 274031 Shandong China

**Keywords:** Lung cancer, Patient prognosis, Six-transmembrane epithelial antigen of the prostate 1, Six-transmembrane epithelial antigen of the prostate 2, Biomarker

## Abstract

**Purpose:**

Lung cancer is the leading cause of cancer-related mortality. STEAP1 and STEAP2 are overexpressed in various cancers. The purpose of this study was to evaluate the expression and prognostic value of STEAP1 and STEAP2 in patients with lung cancer.

**Methods:**

The mRNA expression and protein expression of STEAP1 and STEAP2 and their prognostic characteristics were examined using Oncomine, GEPIA, and Kaplan-Meier (KM) plotters. The correlation analysis of STEAP1 and STEAP2 gene and protein levels was conducted using GeneMANIA and STRING. KEGG pathway analysis was used to explore the related signal pathways of STEAP 1 and STEAP2. Immunohistochemical methods were used to compare the expression of STEAP2 in normal lung and non-small cell lung cancer (NSCLC) tissues. Real-time quantitative polymerase chain reaction, western blotting, and immunocytochemistry were used to evaluate the expression of STEAP1 and STEAP2 in three lung cancer cell lines and normal lung epithelial cell lines.

**Results:**

Analysis of the Oncomine database and GEPIA showed that STEAP1 was upregulated and STEAP2 was downregulated in lung cancer tissue, and both expressions were related to the clinical stage of lung cancer. Immunohistochemical analysis showed that STEAP1 protein expression was significantly upregulated in lung cancer compared to that in adjacent tissues. The expression of STEAP1 was positively correlated with the migration and invasion abilities of lung cancer cells. Compared with paracancer tissues, the expression of STEAP2 protein in lung cancer was significantly downregulated and was correlated with the histological grade of squamous cell carcinoma, pathological classification of adenocarcinoma, tumor, lymph node, and metastasis clinical stage, and lymph node metastasis. The expression of STEAP2 was negatively correlated with the migration and invasion abilities of lung cancer cells. The KM curve showed that the downregulation of STEAP1 expression and upregulation of STEAP2 expression were related to a good lung cancer prognosis.

**Conclusion:**

STEAP1 and STEAP2 are expected to be potential diagnostic and prognostic markers for lung cancer, which may provide more accurate prognostic indicators for lung cancer.

## Introduction

Lung cancer is the main cause of cancer-related mortality (accounting for 18.0% of all cancer-related deaths) [1]. There are 2.2 million new cancer cases and 1.8 million deaths attributed to lung cancer annually and was the second most frequently diagnosed cancer in 2020. Despite progress in early detection and standard treatment, most patients are still diagnosed at an advanced stage of the disease and have a poor prognosis [2]. Among lung cancer patients diagnosed between 2010 and 2014 in most countries, the survival rate in the first 5 years after diagnosis was only 10 to 20% [3]. Chemotherapy, surgery, and radiotherapy are the traditional treatment methods for lung cancer [4]; however, chemotherapy and targeted therapy are broadly drug-resistant [5, 6], and the recurrence of lung cancer in the early postoperative period remains very high [7]. Tumor markers can provide important predictive or prognostic information for the treatment of lung cancer. Therefore, to accurately diagnose the disease and predict prognosis, it is very important to identify new tumor markers for lung cancer.

The 6-transmembrane epithelial antigen of the prostate (STEAP) protein family contains at least five homologous members (STEAP1–4 and STEAP1B), which have been confirmed to be involved in many biological processes [8]. The STEAP family interacts with various genes involved in the cell cycle, thus regulating the growth and proliferation of cancer cells [9]. Recent studies have shown that STEAP participates in intercellular communication through molecular transport, acts as a channel protein or transporter, and may play a role in cell adhesion [10].

STEAP1 is the first member of the STEAP family to be identified. The STEAP1 gene is located on chromosome 7q21.13 and contains 10.4 KB, 5 exons, and 4 introns [11]. STEAP1 is an ion channel or transporter that plays a role in cell adhesion and may promote tumor proliferation and invasion by regulating ion concentrations such as Na +, K +, Ca 2+, and small molecules [4]. STEAP1 is highly expressed in human prostate cancer and is upregulated in a variety of cancers, including lung, bladder, colon, ovarian, and Ewing cancers [12].

The *STEAP2* gene, also known as *STAMP1*, is located on chromosome 7q21.13 and is situated near *STEAP1* and *STEAP4*. It consists of six exons and five introns, encoding 490 amino acids [13]. In addition, the expression of STEAP2 in breast cancer tissues and cells has been reported to be downregulated. STEAP2 has also been found to be overexpressed in other human cancers, such as bladder, colon, pancreas, ovary, testis, and cervical, and Ewing’s sarcoma [14].

Many studies have confirmed changes in STEAP expression patterns in many cancers. This suggests that the STEAP family may be an important therapeutic target in a variety of cancers. STEAP1 and STEAP2, as members of the STEAP family, were initially identified as important metal reductases in vivo and play an important role in maintaining iron homeostasis. The survival of cancer cells is promoted by two main mechanisms: increasing growth and proliferation and inhibiting apoptosis of cancer cells [8]. Previous studies have found that STEAP1 and STEAP2 are associated with poorer patient outcomes through comprehensive microarray screening of bone marrow aspirates in Ewing’s sarcoma patients. And STEAP1 and STEAP2 have previously been reported as potential markers, especially for aggressive prostate cancer [15]. However, the current research on STEAP2 is still in the preliminary stage, and few studies have focused on the prognostic value of STEAP1 and STEAP2 in lung cancer. Therefore, the purpose of this study was to investigate the expression of STEAP1 and STEAP2 and their potential prognostic value in order to provide a basis for new strategies for the treatment of lung cancer.

## Materials and methods

### Oncomine analysis

We used Oncomine (https://www.Oncomine.org) [16], a publicly accessible online database of cancer gene expression profiles, to retrieve STEAP1 and STEAP2 mRNA expression in various cancers.

### Kaplan-Meier (KM) plotter analysis

KM plotter (http://kmplot.com/analysis/) includes the survival data of patients with breast cancer (6234 cases), ovarian cancer (2190 cases), lung cancer (3452 cases), and gastric cancer (1440 cases) [17]. The KM plotter was used to evaluate the expression of STEAP1 and STEAP2 and their relationship with the prognosis of lung cancer patients.

### GEPIA dataset analysis

Gene expression profile interactive analysis (GEPIA) (http://gepia.cancer-pku.cn/) provides fast and customizable functionality based on TCGA and GTEx data [18]. This investigates the correlation between STEAP1 and STEAP2 expression and the relationship between STEAP1 and STEAP2 and the stage of lung cancer.

### GeneMANIA analysis

The interaction between STEAP1 and STEAP2 at the gene level was analyzed using GeneMANIA (http://genemania.org) [19], a network tool for identifying intra-genomic associations.

### STRING analysis

STRING (https://string-db.org/) is a database used to search for the physical interactions between proteins and the functional correlations between proteins. We used STRING to analyze the protein correlations between STEAP1 and STEAP2.

### KEGG pathway analysis

KEGG (http://www.kegg.jp/ or http://www.genome.jp/kegg/) is an encyclopedia of genes and genomes. The main objective of the KEGG database project is to assign functional meaning to genes and genomes at the molecular and higher levels. We used the KEGG database to explore the related signaling pathways of STEAP 1 and STEAP2 [20].

### Cell lines

In this study, the human lung adenocarcinoma cell lines A549 and H1299 and the human large cell lung cancer cell lines H460 and the normal lung epithelial cell line BEAS-2B were used. All chemicals were purchased from Shanghai Institute of Biological Sciences, Chinese Academy of Sciences. The A549, H460, and H1299 cell lines were grown in RPMI-1640 (Sigma Aldrich, St Louis, MO, USA) containing 10% fetal bovine serum (FBS; Clark Bioscience, Richmond, VA, USA) and 1% penicillin-streptomycin solution (Merck, Kenilworth, NJ, USA). BEAS-2B cells were cultured in Dulbecco’s modified Eagle medium supplemented with 10% FBS and 1% penicillin-streptomycin solution and maintained in a 5% CO_2_ atmosphere at 37°C.

### Tumor tissue samples

After obtaining informed consent from the patients, lung tissue samples were collected from the Shandong Provincial Hospital and Qilu Hospital (Shandong, China). Two pathologists examined all specimens. A total of 298 samples were obtained, including 40 normal lungs, 133 squamous cell carcinomas, and 125 adenocarcinoma samples. All lung cancer patients were diagnosed based on the tumor, lymph node, and metastasis (TNM) staging system, and no treatment was performed before tissue samples were collected. This study was approved by the Medical Ethics Committee of Shandong University. All methods were performed in accordance with relevant standards and regulations.

### Transwell migration and invasion assay

#### Transwell chamber invasion experiment

Matrigel was diluted with serum-free medium, and 50 μL was evenly coated on the upper chamber filter membrane of the Transwell chamber. Cells in each group were digested with trypsin and resuspended in a serum-free medium. A 200-μL cell suspension was added to the upper chamber, and 10% FBS was added to the lower chamber. After incubation at 37°C and 5% CO_2_ for 24 h, the cells on the upper layer of the filter membrane were removed, and the cells on the lower surface were fixed with 4% paraformaldehyde. After staining with crystal violet, the cells were dehydrated and sealed. The cells were counted in five random fields to quantify cell invasion ability.

#### Transwell chamber movement experiment

The filter membrane of the upper chamber of the Transwell chamber was not coated with Matrigel, and the other steps were the same as in the Transwell chamber invasion experiment described above.

### Immunohistochemistry

Tissues were fixed using 4% formaldehyde, embedded in paraffin for sectioning, and removed using xylene for 5 min. The samples were then rehydrated using a graded series of alcohol dilutions. Antigen recovery was performed in citrate buffer at 125°C in a steam pressure cooker for 2 min. *Streptomyces* biotin protein-peroxidase staining was performed according to the manufacturer’s instructions and summarized as follows: STEAP1 antibody (1:400 dilution in phosphate-buffered saline [PBS]; cat. no. ab207914, Abcam, Cambridge, UK); STEAP2 antibody (1:100 dilution in phosphate-buffered saline [PBS]; cat. no. ab207914, Abcam, Cambridge, UK); incubation at 4°C overnight; addition of a secondary antibody (Conway Century Company) for 30 min; DAB color; hematoxylin re-dyeing; dehydration seal; and observation of tissue samples using an optical microscope. The staining results were evaluated and graded according to the ratio of staining intensity and the proportion of positive cells in tissue sections or cell climbing slices. Dyeing intensity was scored as follows: 0, no staining; 1, low strength; 2, medium strength; and 3, high strength. Positive cell ratio was divided as follows: 0% 0, 1–25% 1, 26–50% 2, 51–75% 3, and 76–100% 4. The total score was represented by the sum of the staining intensity score and positive cell ratio score (0–7), with a total score ≤ 3 indicating low expression and ≥ 4 indicating high expression. All tissue sections and cell climbing results were blindly evaluated by two researchers. Differences in scoring were resolved through discussion.

### Immunocytochemistry

Cultured cell lines were treated with trypsin and centrifuged to recover the cells. The cells were inoculated into 24-well plates containing cell slides and cultured in a medium for 24 h at 37°C. After the cells reached 60–80% confluence, they were treated with STEAP1 and STEAP2 for immunocytochemistry, DAB staining, and hematoxylin re-staining, respectively. Staining intensity and percentage of stained cells were observed after dehydration and sealing. The rating standards were determined using immunohistochemical methods.

### Real-time quantitative polymerase chain reaction

RNA was extracted from the cell lines using the TRIzol method using a commercially available kit in accordance with the manufacturer’s instructions. The concentration and purity of RNA were determined using a microplate reader. In a 20-μL reaction system using PrimeScript RT kit with gDNA eraser (Takara Bio Inc., Shiga, Japan), 2 μg of total RNA was used as a template to reverse transcribe the complementary DNA (cDNA). According to the manufacturer’s instructions, a LightCycler 480 system (Applied Biosystems Inc., Waltham, MA, USA; Roche, Inc., Basel, Switzerland) was used for real-time quantitative polymerase chain reaction (RT-qPCR), which was performed in triplicate samples in 96-well plates. The qPCR mixture volume in each well was 20 μL, including 10 μL SYBR Premix EX Taq, 0.4 μL PCR forward primer, 0.4 μL PCR reverse primer, 2 μL cDNA, and 7.2 μL sterile water. Specific primers were designed and synthesized by TaKaRa Biotechnology Co., Ltd. (Japan). Primer sequences included the upstream primer for STEAP1, 5′-ACAAGTTGCTAAACTGGGCATATCA-3′, the downstream primer, 5′-CAGTATTGCCAATCCCACAATTC-3′; STEAP2, 5′-CGCTATGGTCCATGTTGCCTA-3, downstream primer, 5′-CCAAGGCTCATTATGCCAAAG-3, an internal reference ACTB upstream primer, 5-TGGCACCCAGCACAATGAA-3, and downstream primer 5-CTAAGTCATAGTCCGCCTAGAAGCA-3′. The experiment was repeated three times.

### Western blotting

Cells were collected and lysed on ice to extract protein using radioimmunoprecipitation assay buffer (i.e., “RIPA”) containing 1 mM phenylmethylsulfonyl fluoride. Protein concentration was determined using the bicinchoninic acid assay (i.e., “BCA”) method. Protein samples (30 μg) were separated using 10% sodium dodecyl sulfate-polyacrylamide gel electrophoresis, and the separated protein samples were transferred to PVDF membranes and blocked with 5% bovine serum albumin. The membrane was then incubated with mouse anti-STEAP1 primary antibody (Abcam, cat. no., ab207914) and mouse anti-STEAP2 primary antibody (AbCam, cat. no., ab207914) or anti-β-actin primary antibody at a dilution of 1:2000 overnight at 4°C. The next day, the membrane was incubated with a secondary antibody at room temperature for 1 h, and the substrate was analyzed using an enhanced chemiluminescence assay (Pierce ECL western blotting substrate; Millipore, Inc., Burlington, MA, USA) and Amersham Imager 600 (GE Healthcare, Milwaukee, WI, USA) chemiluminescence models to visualize imprinting. After the membrane was washed with TBST and developed using the ECL method, the gray value was determined using ImageJ version 1.46r (National Institutes of Health, Bethesda, MD, USA) and normalized to the gray value of β-actin.

### Statistical analysis

Statistical analysis was performed using SPSS (version 20.0; IBM Corporation, Armonk, NY, USA). Quantitative data were assessed using analysis of variance. Count data are expressed as percentages. The Pearson chi-squared or Fisher’s exact test was used for between-group comparisons; differences were considered statistically significant at *P* < 0.05.

## Results

### The mRNA and protein expression of STEAP1 and STEAP2 in breast cancer

**Fig. 1:**
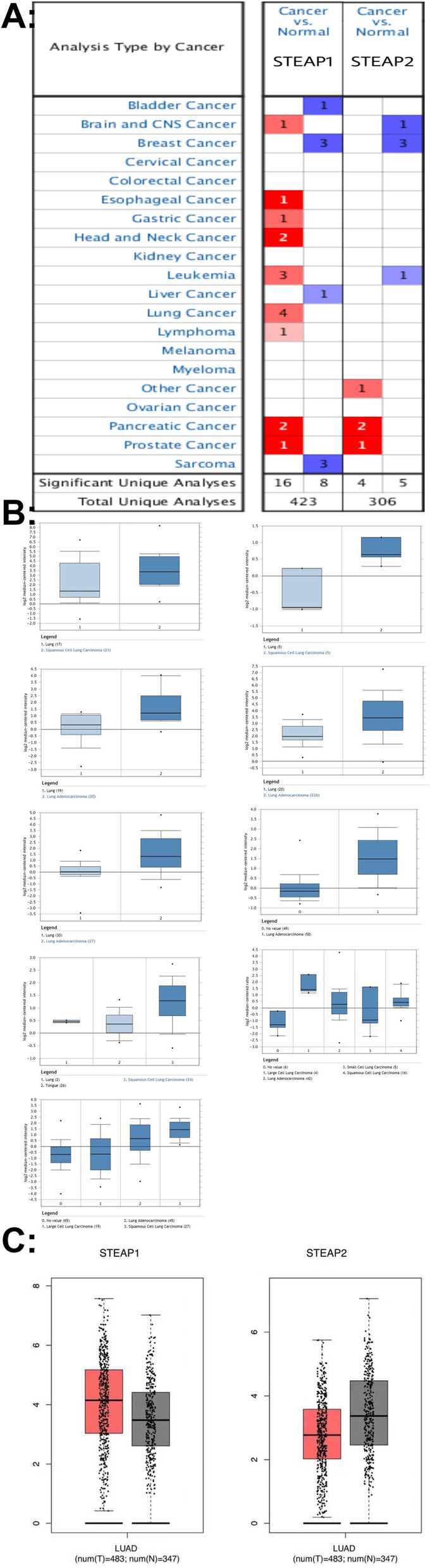
**A** MRNA expression levels of STEAP1 and STEAP2 in different cancer types. Red represents overexpression of the target gene in tumor tissue compared to normal tissue, while blue indicates downregulation of the gene. Color depth indicates the percentage of genes arranged. **B** Based on the Oncomine database, the expression of STEAP1 mRNA in lung cancer tissues was compared with that in normal lung tissues. **C** GEPIA analysis of mRNA expression levels of STEAP1 and STEAP2 in lung cancer. The boxplot of GEPIA gene expression data compared the expressions of STEAP1 and STEAP2 in lung cancer and normal tissues, *P* ≤ 0.05

We used the Oncomine database to analyze the mRNA expression levels of STEAP1 and STEAP2 in various cancers and corresponding normal tissues (Fig. 1A). The results showed that there were 423 and 306 unique analyses for STEAP1 and STEAP2, respectively. STEAP1 expression was upregulated in nine different types of human cancers, including prostate and lung cancer, compared to normal tissues. However, STEAP2 expression is not synchronized across cancers, and there are no data to suggest that STEAP2 expression levels differ in lung cancer.

Oncomine results showed that STEAP1 expression was upregulated in lung cancer. In nine datasets, STEAP1 expression was significantly higher in non-small cell lung cancer (NSCLC) than in normal lung tissue, Lung in Landi Lung and Stearman Lung and Okayama Lung and Su Lung and Hou Lung and Garber Lung and Garber Lung Statistics Adenocarcinoma, Squamous Cell Lung in Hou Lung and Garber Lung and Bhattacharjee Lung and Talbot Lung and Wachi Lung Statistics. It is upregulated in carcinoma, and it is upregulated in large cell lung carcinoma. However, there are insufficient data to show that STEAP2 mRNA levels are significantly different between tumor and normal tissues. All results were statistically significant (Table 1) (Fig. 1B).

**Table 1:** The differential analysis of STEAP1 mRNA expression in Oncomine datasets

Datasets	Lung carcinoma vs. normal	Fold change	*P*
Landi Lung Statistics*	Lung (49) Lung adenocarcinoma (58)	3.033	8.78E−16
Stearman Lung Statistics**	Lung (19) Lung adenocarcinoma (20)	2.888	4.53E−5
Okayama Lung Statistics***	Lung (20) Lung adenocarcinoma (226)	2.703	1.39E−7
Su Lung Statistics****	Lung (30) Lung adenocarcinoma (27)	2.612	7.78E−5
Hou Lung Statistics*****	Lung (65) Lung adenocarcinoma (45)	4.633	5.06E−16
	Lung (65) Squamous cell lung carcinoma (27)	2.451	1.57E−6
Garber Lung Statistics******	Lung (6) Lung adenocarcinoma (42)	2.970	3.89E−4
	Lung (6) Large cell lung carcinoma (4)	7.121	1.47E−4
	Lung (6) Squamous cell lung carcinoma (16)	3.287	2.31E−4
Bhattacharjee Lung Statistics*******	Lung (17) Squamous cell lung carcinoma (21)	2.744	0.019
Talbot Lung Statistics********	Lung (2) Squamous cell lung carcinoma (34)	1.796	2.46E−6
Wachi Lung Statistics*********	Lung (5) Squamous cell lung carcinoma (5)	2.358	0.005

*In Landi Lung Statistics, STEAP1 mRNA expression in lung adenocarcinoma (58) was 3.033 times higher than that in the normal lung (49) (*P* < 0.05). **In Stearman Lung Statistics, STEAP1 mRNA expression in lung adenocarcinoma (20) was 2.888 times higher than that in the normal lung (19) (*P* < 0.05). ***In Okayama Lung Statistics, STEAP1 mRNA expression in lung adenocarcinoma (226) was 2.703 times higher than that in the normal lung (20) (*P* < 0.05). ****In Su Lung Statistics, STEAP1 mRNA expression in lung adenocarcinoma (27) was 2.612 times higher than that in the normal lung (30) (*P* < 0.05). *****In Hou Lung Statistics, STEAP1 mRNA expression in lung adenocarcinoma (45) and squamous cell lung carcinoma (27) was 4.633 and 2.451 times higher than those in the normal lung (65), respectively (*P* < 0.05). ******In Garber Lung Statistics, STEAP1 mRNA expression in lung adenocarcinoma (42), large cell lung carcinoma (4), and squamous cell lung carcinoma (16) was 2.970, 7.121, and 3.287 higher than those in the normal lung (6), respectively (*P* < 0.05). *******In Bhattacharjee Lung Statistics, STEAP1 mRNA expression in squamous cell lung carcinoma (21) was 2.744 times higher than that in the normal lung (17) (*P* < 0.05). ********In Talbot Lung Statistics, STEAP1 mRNA expression in squamous cell lung carcinoma (34) was 1.796 times higher than that in the normal lung (2) (*P* < 0.05). *********In Wachi Lung Statistics, STEAP1 mRNA expression in squamous cell lung carcinoma (5) was 2.358 times higher than that in the normal lung (5) (*P* < 0.05)

We also compared STEAP1 and STEAP2 transcriptional levels between lung cancer and normal tissues using GEPIA. We found that STEAP1 was upregulated in tumor tissues, while the expression level of STEAP2 was significantly downregulated (Fig. 1C).

**Fig. 2:**
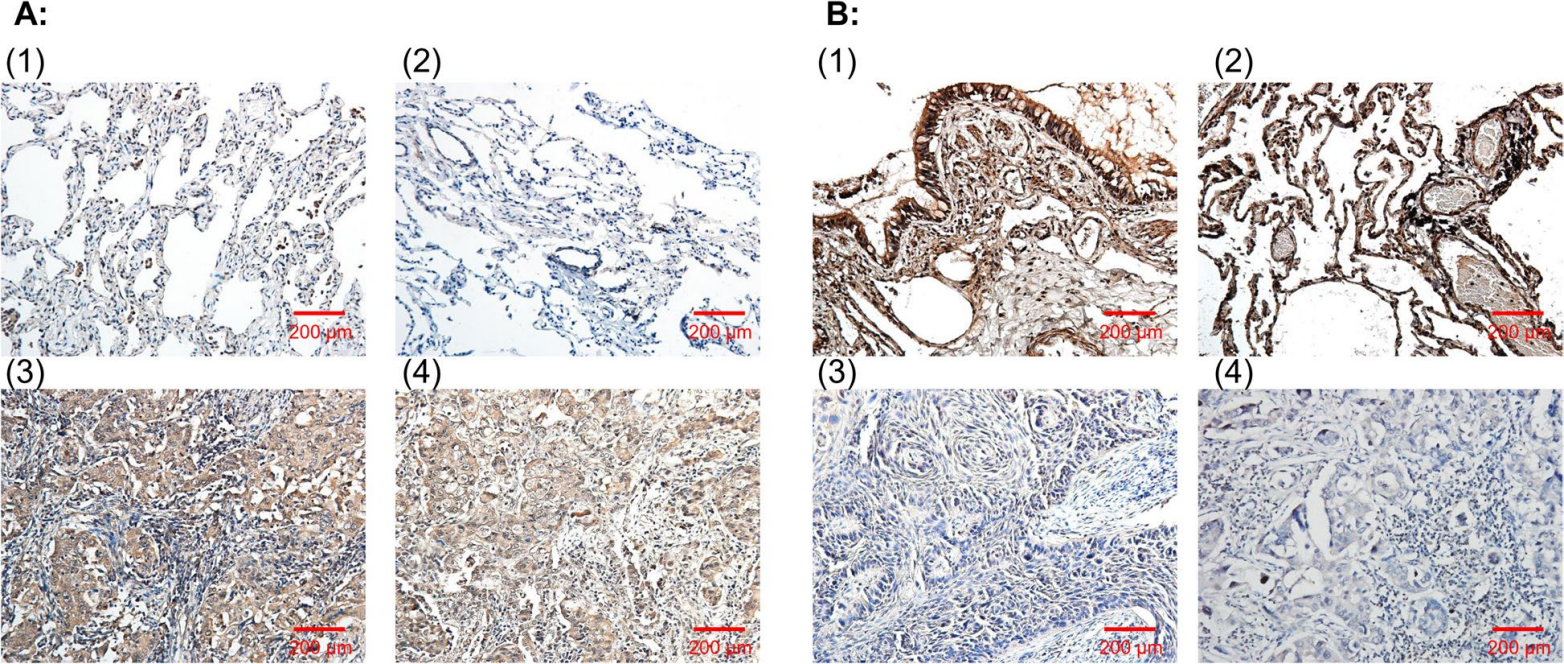
Immunohistochemical staining of adjacent normal lung tissue and lung cancer tissue. **A** Expression of STEAP1 in lung tissues (SP staining, 200×): (1), (2): expression in normal lung tissues; (3): expression in lung squamous cell carcinoma; (4): expression in lung adenocarcinoma. **B** Expression of STEAP2 in lung tissues (SP staining, 200×): (1), (2): expression in normal lung tissues; (3): expression in lung squamous cell carcinoma; (4): expression in lung adenocarcinoma

Immunohistochemistry revealed that the expression of STEAP1 in normal lung tissues was significantly higher than that in NSCLC tissues, and STEAP2 expression in normal lung tissues was significantly lower than that in NSCLC tissues. The overexpression probability of STEAP1 in normal tissues was 12.5% (5/40), which was significantly lower than that in NSCLC tissues (85.7% [221/258]), and the difference was statistically significant (*P* < 0.01) (Fig. 2A) (Table 2). The overexpression probability of STEAP2 in normal tissues was 82.5% (33/40), which was significantly higher than that in NSCLC tissues (16.7% [43/258]), and the difference was statistically significant (*P* < 0.01) (Fig. 2B) (Table 2).

**Table 2:** Expression of STEAP1 and STEAP2 in lung normal and cancer tissues

Pattern of tissue	*N*	STEAP1		*χ*^2^	*P*
		High expression	Low expression		
		*n* (%)	*n* (%)		
Normal lung tissues NSCLC	40 258	5 (12.5) 221 (85.7)	35 (87.5) 37 (14.3)	101.156	0.000
		STEAP2			
Pattern of tissue	*N*	High expression	Low expression	*χ*^2^	*P*
		*n* (%)	*n* (%)		
Normal lung tissues	40	33 (82.5)	7 (17.5)	78.999	0.000
NSCLC	258	43 (16.7)	215 (83.3)		

**Fig. 3:**
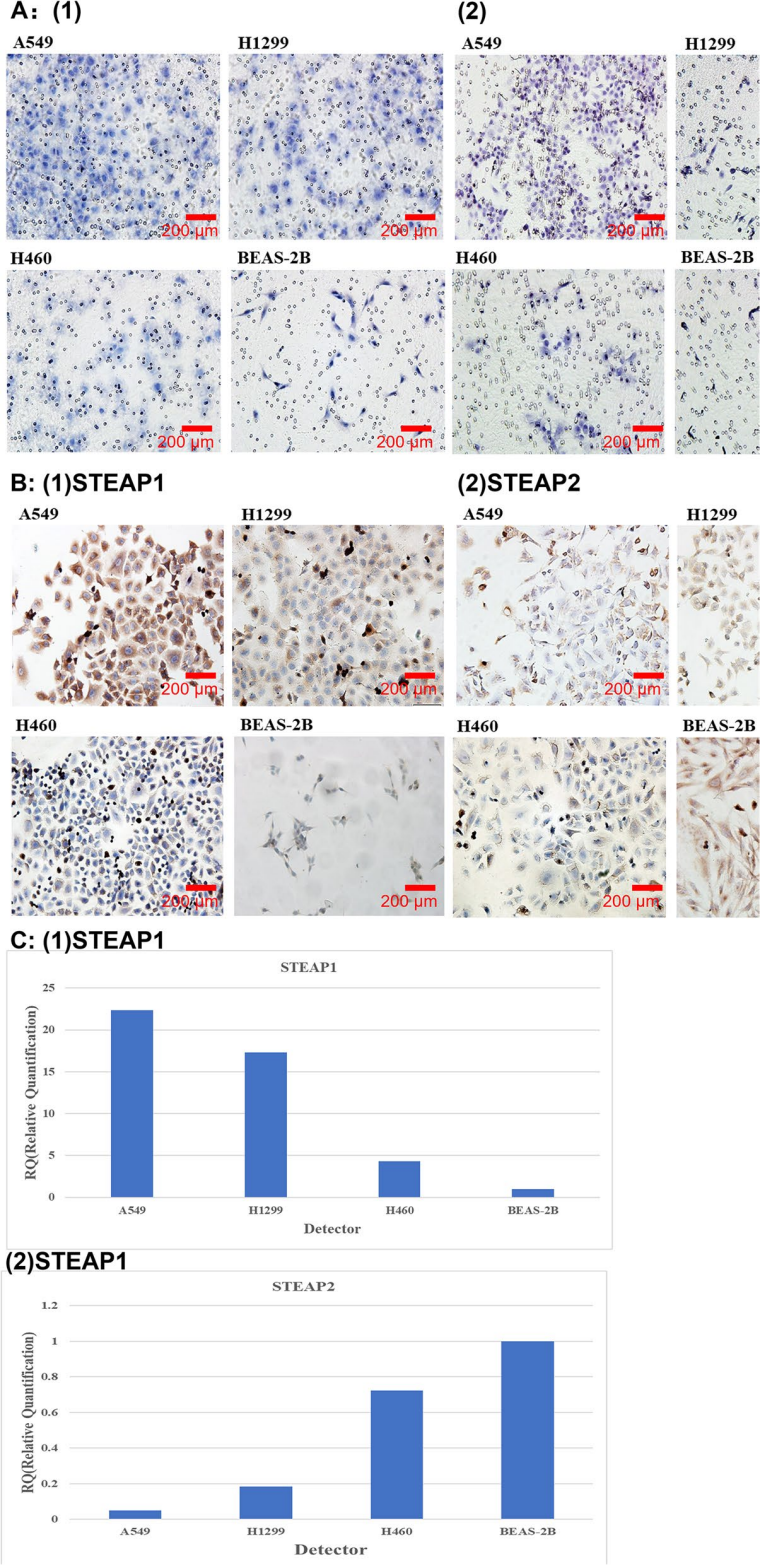
**A** (1) Determination of migration of A549, H1299, H460, and BeAS-2B. (2) Assays of A549, H1299, H460, and BEAS-2B. **B** The expression of STEAP1 and STEAP2 in A549, H1299, H460, and BEAS-2B was determined by immunocytochemistry. **C** Q-PCR was performed to quantitatively detect the mRNA expressions of STEAP1 and STEAP2 in A549, H1299, H460, and BeAS-2B. **D** Western blot analysis was performed to evaluate the expression of STEAP1 and STEAP2 in A549, H1299, H460, and BEAS-2B. β-actin protein was used as an internal positive control

The invasion and migration ability of human lung cancer cell lines A549, H1299, and H460 and the normal lung epithelial cell line BEAS-2B were determined using the Transwell invasion-migration assay. Results revealed that A549 lung cancer cells had the highest invasion and migration abilities, and most cells penetrated the matrix and the filter membrane. At the same time, normal lung epithelial cells, BEAS-2B, had low invasive mobility and weak penetration, while the lung cancer cell lines H1299 and H460 had weaker invasive mobility than A549, but stronger than BEAS-2B (Fig. 3A).

Immunocytochemistry (Fig. 3B), RT-qPCR (Fig. 3C), and western blotting (Fig. 3D) revealed that the expression of STEAP1 in the three lung cancer cell lines (A549, H460, and H1299) was significantly higher than that in the normal lung epithelial cell line BEAS-2B; the expression of STEAP2 in the three lung cancer cell lines (A549, H460, and H1299) was significantly lower than that in the normal lung epithelial cell line BEAS-2B. The expression of STEAP1 was highest in the highly invasive cell line A549. The expression of STEAP1 decreased with a decrease in cell invasion ability, and the lowest expression was found in the normal lung epithelial cell line BEAS-2B. The expression of STEAP2 was the lowest in the highly invasive cell line A549. The expression of STEAP2 increased with a decrease in cell invasion ability, and the highest expression was found in the normal lung epithelial cell line BEAS-2B. The expression of STEAP1 and STEAP2 in lung cancer cells was consistent with that in tissues.

### Relationship between STEAP1 and STEAP2 expression and clinicopathological features in patients with NSCLC

**Fig. 4:**
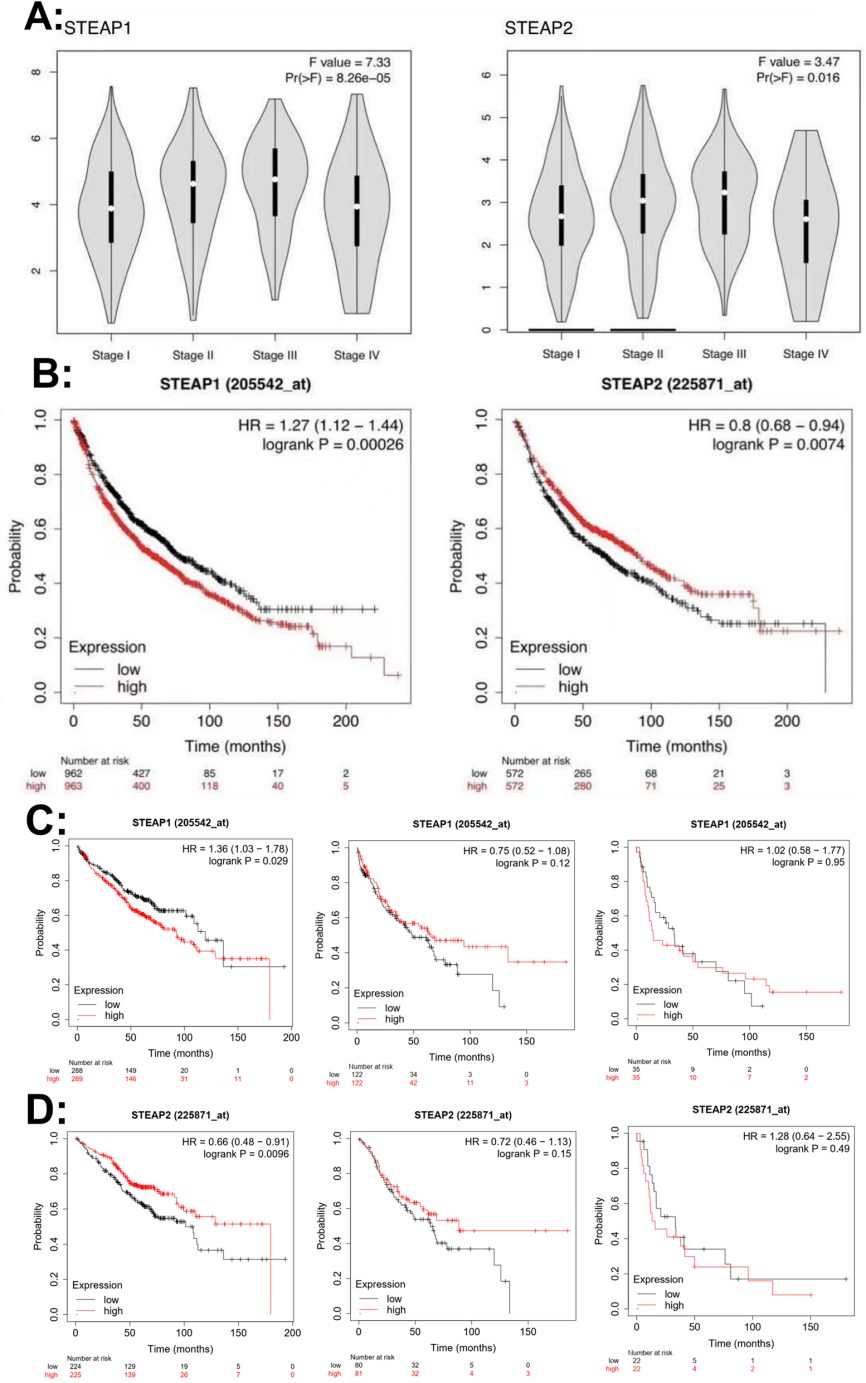
**A** Correlation between the expression of STEAP1 and STEAP2 and tumor stage in lung cancer patients. The expression of STEAP1 and STEAP2 were correlated with the pathological stage of lung cancer (*P* < 0.05). **B** In the Kaplan-Meier plotter database, the high expression of STEAP1 was associated with poor prognosis in lung cancer patients, and the low expression of STEAP2 was associated with poor prognosis in lung cancer patients. **C** In the Kaplan-Meier plotter database, the high expression of STEAP1 in stage I lung cancer patients was associated with poor prognosis, which was statistically significant. STEAP1 expression and prognosis were not significantly different in stage II and III lung cancer patients. D In the Kaplan-Meier plotter database, the low expression of STEAP2 in stage I lung cancer patients was associated with poor prognosis, which was statistically significant. STEAP2 expression and prognosis were not significantly different in stage II and III lung cancer patients

In addition, we used GEPIA to analyze the relationship between STEAP1 and STEAP2 mRNA levels and lung cancer staging. The results showed that STEAP1 and STEAP2 expression was correlated with tumor stage (Fig. 4A). These data suggest that STEAP1 and STEAP2 may play important roles in the tumorigenesis and progression of lung cancer.

The expression level of STEAP1 was not associated with histological NSCLC subtypes, such as squamous cell carcinoma, adenocarcinoma, and adenosquamous carcinoma (*P* > 0.05). The expression of STEAP1 in highly differentiated squamous cell carcinoma is significantly lower than that in poorly differentiated squamous cell carcinoma. The expression of STEAP1 in lepidic adenocarcinoma (highly differentiated) was significantly lower than that in solid adenocarcinoma and micropapillary adenocarcinoma (poorly differentiated) (*P* < 0.05). Low STEAP1 expression was positively correlated with high clinical stage and positive lymph node metastasis in patients (Tables 3 and 4). The expression level of STEAP2 was not associated with histological NSCLC subtypes, such as squamous cell carcinoma, adenocarcinoma, and adenosquamous carcinoma (*P* > 0.05). However, it was associated with the histological grade of squamous cell carcinoma, pathological classification of adenocarcinoma, TNM clinical stage, and lymph node metastasis (*P* < 0.05). The expression of STEAP2 in highly differentiated squamous cell carcinoma was significantly higher than that in poorly differentiated squamous cell carcinoma. The expression of STEAP2 in lepidic adenocarcinoma (highly differentiated) was significantly higher than that in solid adenocarcinoma and micropapillary adenocarcinoma (poorly differentiated) (*P* < 0.05). Low STEAP2 expression was negatively correlated with high clinical stage and positive lymph node metastasis in patients (Tables 3 and 4).

**Table 3:** The relationship between the expression of STEAP1 and clinicopathological features of NSCLC patients

Pathological features	*N*	STEAP1		*χ*^2^	*P*
		High expression	Low expression		
		*n* (%)	*n* (%)		
**Age**				2.721	0.099
≤60	130	116 (89.2)	14 (10.8)		
>60	128	105 (82.0)	23 (18.0)		
**Sex**				0.433	0.510
Male	159	138 (86.8)	21 (13.2)		
Female	99	83 (83.8)	16 (16.2)		
**TNM clinical stages**				23.668	0.000
I and II stages	108	79 (73.1)	29 (26.9)		
III and IV stages	150	142 (94.7)	8 (5.3)		
**Histological type**				0.520※	0.974※
Squamous carcinoma	121	103 (85.1)	18 (14.9)	11.140#	0.004#
I grade	32	18 (56.3)	14 (43.7)		
II grade	48	36 (75.0)	12 (25.0)		
III grade	41	37 (90.2)	4 (9.8)		
Adenocarcinoma	112	96 (85.7)	16 (14.3)	13.684*	0.008*
Lepidic-predominant	25	13 (52.0)	12 (48.0)		
Acinar-predominant	21	13 (61.9)	8 (38.1)		
Papillary-predominant	19	12 (63.2)	7 (36.8)		
Solid-predominant	24	21 (87.5)	3 (12.5)		
Micropapillary-predominant	23	21 (91.3)	2 (8.7)		
Adenosquamous carcinoma	25	21 (84.0)	4 (16.0)		
**Lymphatic metastasis**				32.066	0.000
N0	95	66 (69.5)	29 (30.5)		
N+	163	155 (95.1)	8 (4.9)		

※There was no statistical significance in the high expression rate of STEAP1 in different pathological types of NSCLC, such as squamous cell carcinoma, adenocarcinoma, and adenosquamous carcinoma (*P* > 0.05). #There was statistical significance in the high expression rate of STEAP1 in different histological grades of squamous cell carcinoma, when the histological grade increased, the high expression rate of STEAP1 increased (*P* < 0.05). *There was statistical significance in the high expression rate of STEAP1 in different pathological subtypes of adenocarcinoma; the high expression rate of STEAP1 in well-differentiated lepidic adenocarcinoma was significantly lower than that in poorly differentiated solid and micropapillary adenocarcinoma (*P* < 0.05)

**Table 4:** The relationship between the expression of STEAP2 and clinicopathological features of NSCLC patients

Pathological features	*N*	STEAP2		*χ*^2^	*P*
		High expression	Low expression		
		*n* (%)	*n* (%)		
**Age**				0.198	0.656
≤60	130	23 (17.7)	107 (82.3)		
>60	128	20 (15.6)	108 (84.4)		
**Sex**				0.030	0.864
Male	159	27 (17.0)	132 (83.0)		
Female	99	16 (16.2)	83 (83.8)		
**TNM clinical stages**				19.379	0.000
I and II stages	108	31 (28.7)	77 (71.3)		
III and IV stages	150	12 (8.0)	138 (92.0)		
**Histological type**				0.230※	0.891※
Squamous carcinoma	121	20 (16.5)	101 (83.5)	9.408#	0.009#
I grade	32	13 (40.6)	19 (59.4)		
II grade	48	13 (27.1)	35 (72.9)		
III grade	41	4 (9.8)	37 (90.2)		
Adenocarcinoma	112	18 (16.1)	94 (83.9)	14.298*	0.006*
Lepidic-predominant	25	12 (48.0)	13 (52.0)		
Acinar-predominant	21	9 (42.9)	12 (57.1)		
Papillary-predominant	19	6 (31.6)	13 (68.4)		
Solid-predominant	24	3 (12.5)	21 (87.5)		
Micropapillary-predominant	23	2 (8.7)	21(91.3)		
Adenosquamous carcinoma	25	5 (20.0)	20 (80.0)		
**Lymphatic metastasis**				35.352	0.000
N0	95	33 (34.7)	62 (65.3)		
N+	163	10 (6.1)	153 (93.9)		

※There was no statistical significance in the high expression rate of STEAP2 in different pathological types of NSCLC, such as squamous cell carcinoma, adenocarcinoma, and adenosquamous carcinoma (*P* > 0.05). #There was statistical significance in the high expression rate of STEAP2 in different histological grades of squamous cell carcinoma, when the histological grade increased, the high expression rate of STEAP2 decreased (*P* < 0.05). *There was statistical significance in the high expression rate of STEAP2 in different pathological subtypes of adenocarcinoma; the high expression rate of STEAP2 in well-differentiated lepidic adenocarcinoma was significantly higher than that in poorly differentiated solid and micropapillary adenocarcinoma (*P* < 0.05)

### Relationship between STEAP1 and STEAP2 expression and prognosis in NSCLC patients

The KM plotter database was used to analyze the relationship between the expression of STEAP2 and the prognosis of patients with NSCLC. The results revealed that the prognosis of lung cancer patients with low STEAP1 expression was significantly better than that of lung cancer patients with a high expression of STEAP1 (hazard ratio [HR] 1.27 [95% confidence interval 1.12–1.44]; log-rank *P* = 0.00026) and STEAP1, which therefore suggested poor patient prognosis. The prognosis of lung cancer patients with high expression of STEAP2 was significantly better than that of lung cancer patients with a low expression of STEAP2 (*HR* 0.8 [95% confidence interval 0.68–0.94]; log-rank *P* = 0.0074) and STEAP2, suggesting poor patient prognosis. Specifically, the high expression of STEAP1 in patients with stage I lung cancer was associated with poor prognosis, and the low expression of STEAP2 in patients with stage I lung cancer was associated with poor prognosis, both of which were statistically significant (*P* < 0.05). The expression and prognosis of STEAP1 and STEAP2 in patients with stage II and III lung cancer were not statistically significant (*P* >0.05). The results are shown in Fig. 4B–D.

### Correlation analyses of STEAP1 and STEAP2 in lung cancer patients

**Fig. 5:**
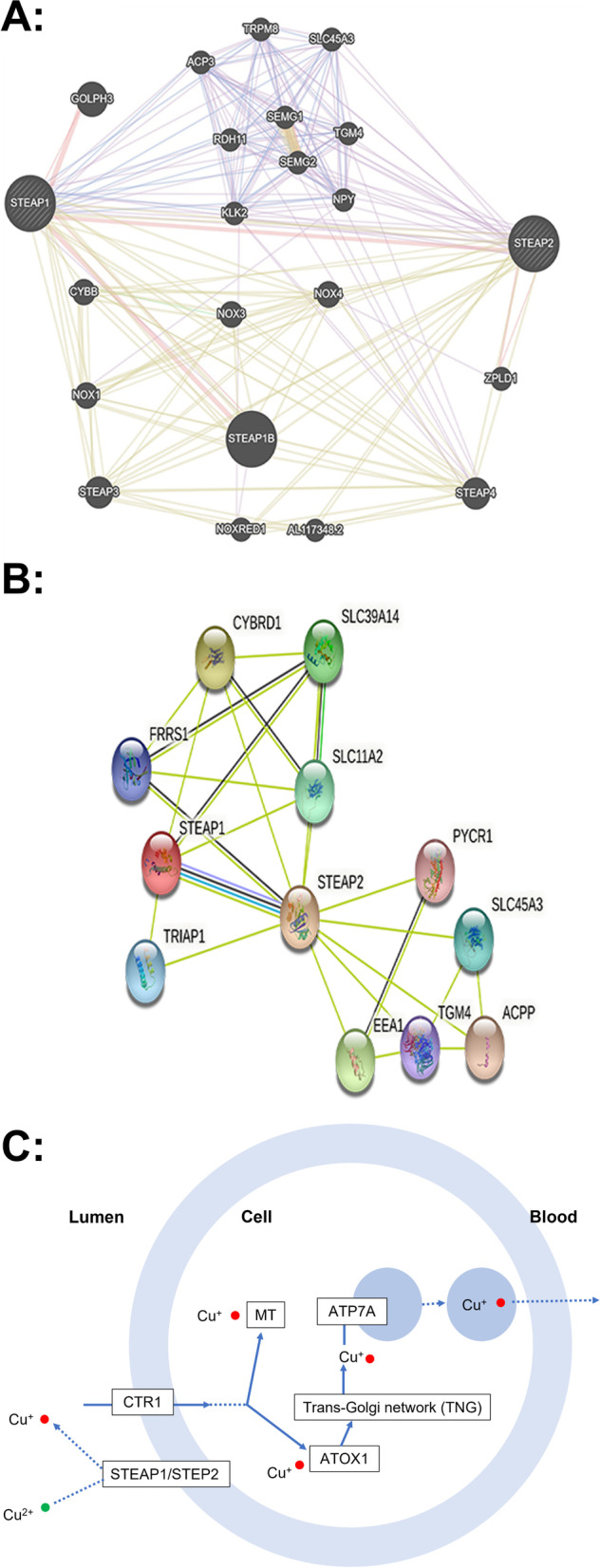
STEAP1 and STEAP2 interactions at gene and protein levels in lung cancer patients. **A** Gene-gene interaction network between STEAP1 and STEAP2 in the GeneMANIA dataset. **B** Protein-protein interaction network between STEAP1 and STEAP2 in the STRING dataset. **C** STEAP1 and STEAP2 involve pathways based on KEGG analysis and are expected to be involved in mineral absorption

We used GeneMANIA to analyze the relationship between STEAP1 and STEAP2 at the gene level (Fig. 5A). The results showed that STEAP1 and STEAP2 shared protein domains, as well as physical interactions. In addition, we identified the interaction between STEAP1 and STEAP2 at the protein expression level using STRING (Fig. 5B). STEAP1 has been shown to interact with STEAP2 in text mining, protein homology, and co-expression. Through KEGG pathway analysis, the important role of STEAP1 and STEAP2 in normal pathological processes has been confirmed by mineral absorption (Fig. 5C). We found that STEAP1 expression was negatively correlated with STEAP2.

## Discussion

This study showed that STEAP1 protein overexpression was significantly correlated with poor prognosis of lung cancer, and STEAP2 protein downregulation was significantly correlated with poor prognosis of lung cancer. Although abnormal expression of the STEAP family has been reported in a variety of cancers, the expression and prognostic value of STEAP2 in lung cancer remains unclear. We adopted bioinformatics methods to systematically and comprehensively analyze the expression level and possible prognosis of STEAP1 and STEAP2 in lung cancer. These results reveal the potential of STEAP1 and STEAP2 as prognostic biomarkers for lung cancer and may be potential targets for future lung cancer therapy.

STEAP1 was the first to be discovered and the smallest member of the STEAP family [21]. STEAP1 has been studied in a variety of cancers, such as breast, prostate, and stomach cancers [7], and has been shown to play a role in tumorigenesis and tumor inhibition [4]. STEAP1 expression is significantly increased in prostate cancer, and silencing STEAP1 expression can inhibit the proliferation of prostate cancer cells and promote cell apoptosis [22]. STEAP1 is upregulated in LUAD and is associated with clinicopathological features and prognosis of patients with LUAD, and STEAP1 expression is associated with LUAD metastasis and EMT. Knockdown STEAP1 significantly inhibited the proliferation and migration of LUAD cells [4]. In this study, we found through an online database that STEAP1 expression level was significantly upregulated in lung cancer tissue, and high STEAP1 expression was positively correlated with poor prognosis. In this regard, we conducted experimental verification of normal lung tissue and lung cancer cases. Compared with normal lung tissue and epithelial cells, the expression of STEAP1 in lung cancer tissue was upregulated. STEAP1 expression was related to the pathological stage, lymph node metastasis, and histological grade of lung cancer, and the expression of STEAP1 gradually increased with the enhancement of the invasion ability of lung cancer cells.

STEAP2 has been reported to be downregulated in breast cancer [23]. STEAP2 overexpression significantly inhibited proliferation and clonogenesis in breast cancer cells. Upregulation of STEAP2 can inactivate the PI3K/AKT signaling pathway and inhibit the proliferation and invasion of breast cancer cells. Upregulation of STEAP2 can inhibit the invasion and metastasis of breast cancer cells by inhibiting epithelial-mesenchymal transformation by affecting transcription factors [14]. In contrast, STEAP2 is significantly overexpressed in prostate cancer, and its overexpression promotes the proliferation, migration, and invasion of tumor cells [23]. Activation of the ERK pathway by STEAP2 leads to partial stagnation of the g0-G1 cell cycle in cancer cells, increasing proliferation and tumor development [24]. In addition, STEAP2 overexpression in normal prostatic epithelial cells increased their migration and invasion ability [14]. STEAP2 has also been found to be overexpressed in other human cancers, including bladder, colon, pancreatic, ovarian, testicular, and cervical cancers and Ewing’s sarcoma [14]. However, there are few studies on the correlation between STEAP2 and other cancers, and there are currently few studies on the correlation between STEAP2 and lung cancer. The results of the online database in this study showed that the expression level of STEAP2 was downregulated, and high STEAP2 expression was positively correlated with a good prognosis. Experimental verification showed that, compared with normal lung tissue and epithelial cells, the expression of STEAP2 was downregulated in lung cancer tissue, and STEAP2 was related to the pathological stage, lymph node metastasis, and histological grade of lung cancer. The expression of STEAP2 decreased gradually with an increase in the invasion ability of lung cancer cells. Our findings provide new evidence for the role of STEAP2 in lung cancer at the molecular level.

Recent studies have reported that the STEAP family, as channel proteins or transporter proteins, may play a role in cell adhesion [3] and are widely expressed in normal human tissues. Its important role in normal pathological processes has previously been demonstrated through mineral absorption, TP53 regulation of cell death genes, and transcription of iron death [23]. In this study, we analyzed STEAP1 and STEAP2 at the gene and protein levels, and the results showed that STEAP1 and STEAP2 had the same protein domain and significant physical interaction, and through KEGG pathway analysis, the important role of STEAP1 and STEAP2 in the normal pathological process was explored, and it was confirmed that both STEAP1 and STEAP2 were involved in mineral absorption process in normal human tissues. STEAP1 is a complete membrane protein with sequence homology to three enzymes (STEAP2–STEAP4) that catalyzes NADPH-dependent iron (III) reduction [25]. STEAP1 lacks the N-terminal NADPH oxidoreductase of the other STEAP [26] and does not show cellular ferric reductase activity [25]; thus, STEAP1 may play a functional role in heterooligomeric complexes with other STEAP para-homologues [27]. A possible candidate for the isodimerization of STEAP1 could be STEAP2, as both proteins are co-purified in detergents, suggesting that they can form functional complexes [25]. Studies have shown that STEAP1 can transfer an electron through the heme group to reduce Fe 3+ to Fe 2+ and Cu 2+ to Cu + to form homologous trimers or heterotrimers with STEAP2 [23], and promote copper absorption, this is consistent with the pathway in our KEGG analysis that STEAP1 and STEAP2 may play a role in cancer by forming functional complexes involved in the process of mineral absorption. These two proteins appear to be significantly co-expressed in cancer entities [26]; however, we found their expression levels in lung cancer were negatively correlated, which countered to our conventional cognition. However, at present, there are relatively many studies on the role of STEAP1 in the occurrence and development of cancer, but the pathogenesis of STEAP2 in lung cancer is still unclear. Therefore, our controversial result that STEAP1 is negatively correlated with STEAP2 expression level in lung cancer may not be a “real” controversy, and further studies are still needed to clarify.

The potential of STEAP1 and STEAP2 as prognostic biomarkers has previously been demonstrated in many other cancers [28]; furthermore, STEAP1 and STEAP2 are reported to be excellent markers for identifying mesenchymal stem cells, which may be by far the most likely Ewing tumor origin cells and are highly sensitive markers [15]. As a potentially valuable marker of lung cancer, previous studies have demonstrated that the identification of STEAP1 mRNA in the serum of cancer patients by highly sensitive and specific real-time PCR can distinguish patients with lung cancer from healthy subjects [11], STEAP1 may be a highly specific biomarker in lung cancer [29], for STEAP2, there are few relevant studies in lung cancer, and to date, no studies have evaluated the co-expression of different STEAP proteins. Compared with the recognized classical markers of lung cancer, STEAP1 and STEAP2 have obvious advantages. Considering the possible association between STEAP1 and STEAP2, STEAP1 and STEAP2 seem to be significantly co-expressed in 59 cancer cell lines [28], but are significantly negatively correlated in lung cancer. Compared with a single prognostic marker, simultaneous assessment of multiple markers may be more accurate for the prognosis of lung cancer. These results suggest that the two may be prognostic markers with high specificity and sensitivity in lung cancer.

In summary, this study focused on the expression of STEAP1 and STEAP2 in lung cancer and evaluated their clinical and prognostic value. The results of previous studies and the analysis results of this study suggest that STEAP1 and STEAP2 may be potential diagnostic markers of lung cancer, which can provide a basis for the prognostic assessment of lung cancer. However, there are some gaps in our research; as such, more studies are needed to elucidate the molecular mechanisms of STEAP1 and STEAP2 in lung cancer.

## Data Availability

The datasets used during the current study are available from the corresponding author on reasonable request.
